# Accuracy of targeted wire-guided tube thoracostomy in comparison with classical surgical chest tube placement: a clinical study

**DOI:** 10.1186/cc14396

**Published:** 2015-03-16

**Authors:** A Protic, I Barkovic, A Sustic

**Affiliations:** 1 University Hospital Rijeka, Croatia

## Introduction

Chest tube malfunction, after the tube thoracostomy, is often the result of an inappropriate chest tube tip position. The aim of this study was to analyze the precision of chest tube placement using the targeted wire guide technique (TWG technique) with a curve dilator and to compare it with the classical surgical technique (CS technique).

## Methods

In this clinical study 80 patients with an indication for thoracic drainage due to pneumothorax or pleural effusion were included. The experimental group contained 39 patients whose chest tube was placed using the TWG technique. The control group contained 41 patients whose chest tube was placed using the CS technique.

## Results

The comparison of the outcomes of the two techniques applied suggests that the TWG technique was significantly more successful, irrespective of patient diagnosis (TWG vs. CS in all patients, 78.4% vs. 36.6%, *P *< 0.001). See Table 1.

## Conclusion

Using a curved dilator and the TWG technique for the thoracic drainage procedure we found statistically significant advantage to the TWG technique in comparison with the CS technique regarding precise chest tube placement within the pleural cavity.

**Table 1:** 

Diagnosis	*N *	CS technique (% of success)	TWG technique (% of success)	*P *value
Pleural effusion	47	37.5	78.2	0.005
Pneumothorax	31	35.3	78.6	0.029
Total	78	36.6	78.4	<0.001
